# Erythrocytes 3D genome organization in vertebrates

**DOI:** 10.1038/s41598-021-83903-9

**Published:** 2021-02-24

**Authors:** Anastasia Ryzhkova, Alena Taskina, Anna Khabarova, Veniamin Fishman, Nariman Battulin

**Affiliations:** 1 Institute of Cytology and Genetics SB RAS, Novosibirsk, Russia; 2 Novosibirsk State University, Novosibirsk, Russia

**Keywords:** Evolutionary biology, Genome, Genomics, Nuclear organization, Haematopoiesis

## Abstract

Generation of mature red blood cells, consisting mainly of hemoglobin, is a remarkable example of coordinated action of various signaling networks. Chromatin condensation is an essential step for terminal erythroid differentiation and subsequent nuclear expulsion in mammals. Here, we profiled 3D genome organization in the blood cells from ten species belonging to different vertebrate classes. Our analysis of contact maps revealed a striking absence of such 3D interaction patterns as loops or TADs in blood cells of all analyzed representatives. We also detect large-scale chromatin rearrangements in blood cells from mammals, birds, reptiles and amphibians: their contact maps display strong second diagonal pattern, representing an increased frequency of long-range contacts, unrelated to TADs or compartments. This pattern is completely atypical for interphase chromosome structure. We confirm that these principles of genome organization are conservative in vertebrate erythroid cells.

## Introduction

Red blood cells (RBC) are the most common type of blood cells in vertebrates, and by some estimates are the most abundant cell type. For example, in humans, they account for about 70% of all cells in the body^1^. One of the key processes during erythrocyte maturation is gradual condensation of their nucleus. In all vertebrates except for mammals, the erythrocyte genome is highly compacted with low to zero transcriptional activity. During mammalian erythropoiesis, this process is especially pronounced, since immature RBC extrude their pyknotic nucleus at the end of their differentiation^2^. Nuclear expulsion is a unique mammalian adaptation enhancing oxygen-carrying capacity and flexibility of erythrocyte. Such dramatic structural changes in the nucleus of differentiating erythroid cells cannot occur without significant 3D reorganization of the erythroid cell genome.

3D architecture of the genome serves an important functional purpose in transcriptional regulation. Genome reorganization upon erythroid differentiation has been comprehensively studied within the biologically well characterized globin loci. Recent high-resolution Capture-C data combined with the polymer physics model show that 3D structure of the globin locus undergoes a significant change, forming a separated domain, with specific enhancer contacts^3^. Moreover, α-globin gene domain forms an interesting spatial pattern: a hairpin structure folded on itself^4^.

In addition to the maintenance of specific physical contacts, regulating transcriptional activity during interphase, 3D genome organization has an important structural role in the mitotic chromatin compaction upon mitotic entry. Recent work indicates that in prophase, the typical interphase organization of chromosomes is lost and a fundamentally new pattern occurs. More specifically, by late promethaphase chromatin compartments and TADs disappear; the Hi-C contact map becomes ‘flat’ and essentially reflects only a decay of contact frequency with genomic distance. Thus, 3D structures, associated with specific genomic motifs, globally disappear. However, when cells enter prometaphase, their Hi-C maps display a second diagonal band, representing an increased frequency of long-range contacts. Polymer modeling revealed that this pattern could be explained if we assume that at the scale of megabases chromosomes are folded through regular periodic interactions: loops emanating from a ‘spiral staircase’ scaffold. Importantly, condensin complex is required for the formation of such helical loop arrangements^5^.

Until recently, the phenomenon of second diagonal was only described for cells in prophase, assuming that this feature of genome organization is unique for the mitotic stage of cell cycle. However, examining the 3D genome organization in chicken erythrocytes, we discover that their contact maps display a second diagonal band at 15–17 Mb distance^6^. Mature avian erythrocytes represent cells in the G1 phase of interphase. Therefore, at least for some cell types, the pattern of second diagonal can be a specific feature of the interphase genome organization.

Here, we explore the 3D genome organization of blood cells from 10 species belonging to different vertebrate classes. We find that chromosome organization in blood cells of all analyzed representatives of different classes is characterized by the absence of TADs. Besides, in mammals, birds, reptiles and amphibians, contact maps of the blood cells display a strong second diagonal pattern, with the position of the band depending on the genome size. Thus, we demonstrate that the described features of the genome organization in the erythroid cells are evolutionary conserved among vertebrates.

## Results

### The key features of the erythrocytes’ genome organization

**Figure 1:**
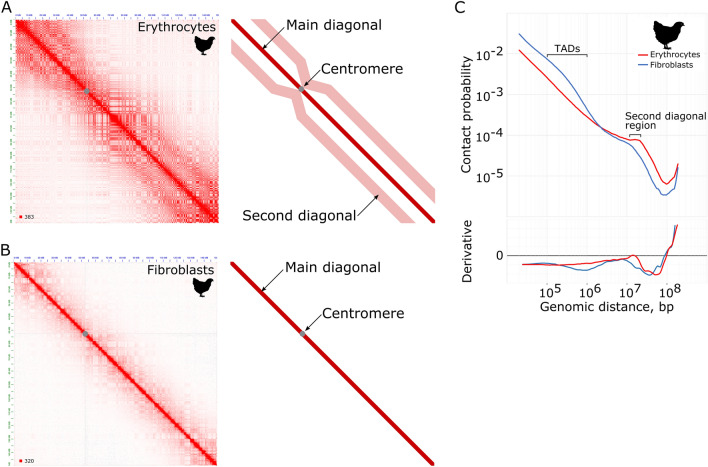
The key features of the erythrocytes’ genome organization on the example of chicken. (**A**) Hi-C contact heatmaps of erythrocytes (left) and a schematic representation of the map with the second diagonal marked (right). (**B**) Hi-C contact heatmaps of fibroblasts (left) and a schematic representation of the map (right). (**C**) The dependence of the contact probability on the genomic distance P(s) averaged over all chromosomes for chicken erythrocytes and fibroblasts. Bottom panel: derivatives of P(s) curves. Note that the shape of the curves differs in TADs typical size region and in the second diagonal region. Hi-C maps were visualized with Juicebox (version 1.11.08)^7^.

Earlier, upon the analysis of the 3D genome organization in birds, we found that chicken erythrocytes appear to have an unusual pattern of genome folding. Figure 1 shows the comparative analysis of Hi-C maps from chicken erythrocytes and chicken fibroblasts. On the whole-chromosome view, contact maps from both cell types display a characteristic checkerboard pattern—a manifestation of the active A- and inactive B- chromatin compartments. However, on the erythrocytes’ heatmap we observed a second diagonal—an increased interaction frequency for genomic loci separated by 15 Mb. In contrast to the contact maps from mitotic cells, which lack chromatin compartments, erythrocyte map simultaneously displays both patterns: the plaid pattern of compartments and the second diagonal, which makes the erythrocyte map very recognizable.

Another characteristic macro-scale feature of genome organization is a probability of chromatin contacts (P) depending on genomic distance between them (s). Analysis of P(s) relationship is a robust statistical approach, generally employed for studying physical models of DNA packaging. P(s) curve follows a general principle where the contact frequency decays with an increase in genomic distance. However, different mechanisms of the 3D genome organization may have various effects on the P(s) curve, changing the shape of a slope or modifying the curvature. On the P(s) curve from erythrocytes data we observed that following a general decay of contact frequencies, a distinct peak of increased contact frequencies at a 10–15 Mb distance appears, corresponding to the second diagonal (Fig. 1C).

Another notable feature of chicken erythrocytes’ genome is the absence of typical TADs. On the P(s) plot, local compaction, corresponding to TADs appears as a shoulder between 10^5^ to 10^6^ bp distance. This slope is readily seen in chicken fibroblasts, while in erythrocytes this region of the curve is completely flat and the drop of contact frequency is linear.

Thus, complete absence of TADs and appearance of the second diagonal, while preserving chromatin compartments, are two characteristic features, making the 3D genome organization of chicken erythrocyte exceptional.

**Figure 2:**
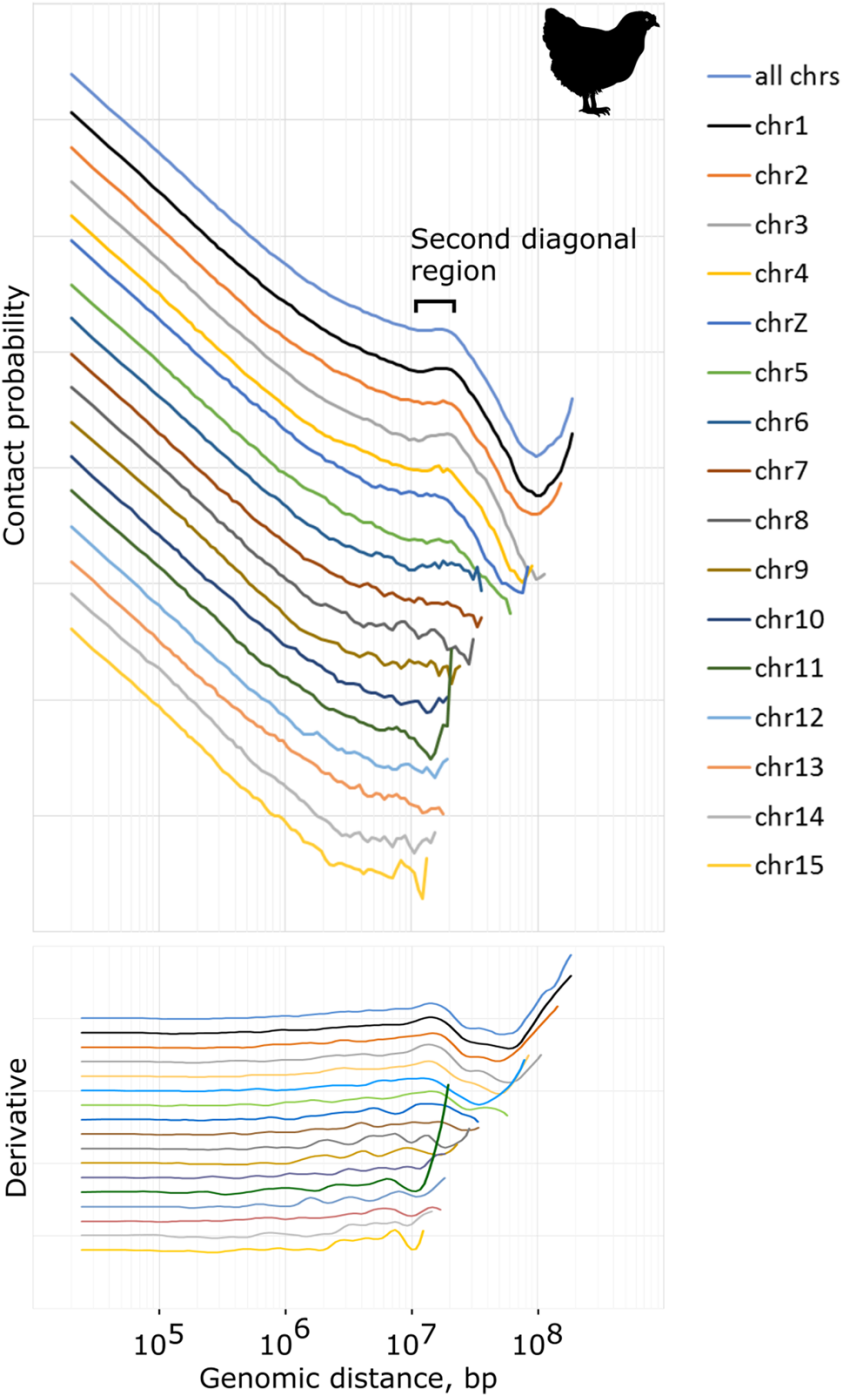
Contact frequency P(s) derived from Hi-C data obtained from chicken erythrocytes. Curves for genome average (all chrs) and long individual chromosomes are shown. The hump of the second diagonal is obvious on the first ~ six longest chromosomes. Bottom panel: derivatives of P(s) curves.

The second diagonal is an intrachromosomal feature. Although it is worth noting, that since chromosomes range in size, the second diagonal is visually more noticeable on longer chromosomes. To understand the positioning of the second diagonal on chromosomes of different size, we plotted the P(s) separately for each chicken chromosome. On the Fig. 2 it is clearly shown that the characteristic peak of frequencies is observed on the six longest chicken chromosomes. Besides, the position of the peak on the curve remains the same. For the chromosomes ~ 20 Mb, or less in size, the increased contact frequency is no longer visible, because the scaffold becomes shorter. Apparently, contacts within one chromosome arm are the most relevant for the analysis (Fig. 1A). However, for most species, we do not have the coordinates of the centromere, so this mode of P(s) construction is not suitable for the analysis of non-model species.

### Hi-C data from erythroid cells of different vertebrate species

We sought to find out, whether the described features of the 3D genome organization in erythrocytes are unique for chicken, or could it be a characteristic chromatin structure of vertebrate RBCs. However, since RBCs make up the vast majority of blood cells, an admixture of other cell types does not have a significant effect on the obtained data, and in fact, a Hi-C map from a blood sample, would be a map from RBCs. Therefore, we used publicly available Hi-C data from blood samples to analyze the 3D organization of the erythrocyte genome in non-mammalian vertebrate classes.

Since in mammals erythroblasts eject their nucleus during maturation, their mature circulating erythrocytes are nuclear-free. However, upon differentiation, mammalian erythroid cells undergo major structural changes, including condensation of chromatin and inhibition of transcriptional activity. This process is common to all vertebrates. Based on this, for the analysis of mammalian erythroid cells, we used the last nucleated stages of maturation. Data for mouse erythroblasts were obtained during this study. Using fluorescence-activated cell sorting (FACS), we isolated cells at poly-/orthochromatic stages of differentiation directly from mouse bone marrow. We have additionally analyzed previously published data for the in vitro differentiated human erythroid progenitors. As far as we know, there is no published Hi-C data for human poly-/orthochromatic erythroblasts. Although in Huang et al. the authors have studied the genomic organization of earlier precursors of an erythroid lineage, namely basophilic erythroblasts^8^.

**Figure 3:**
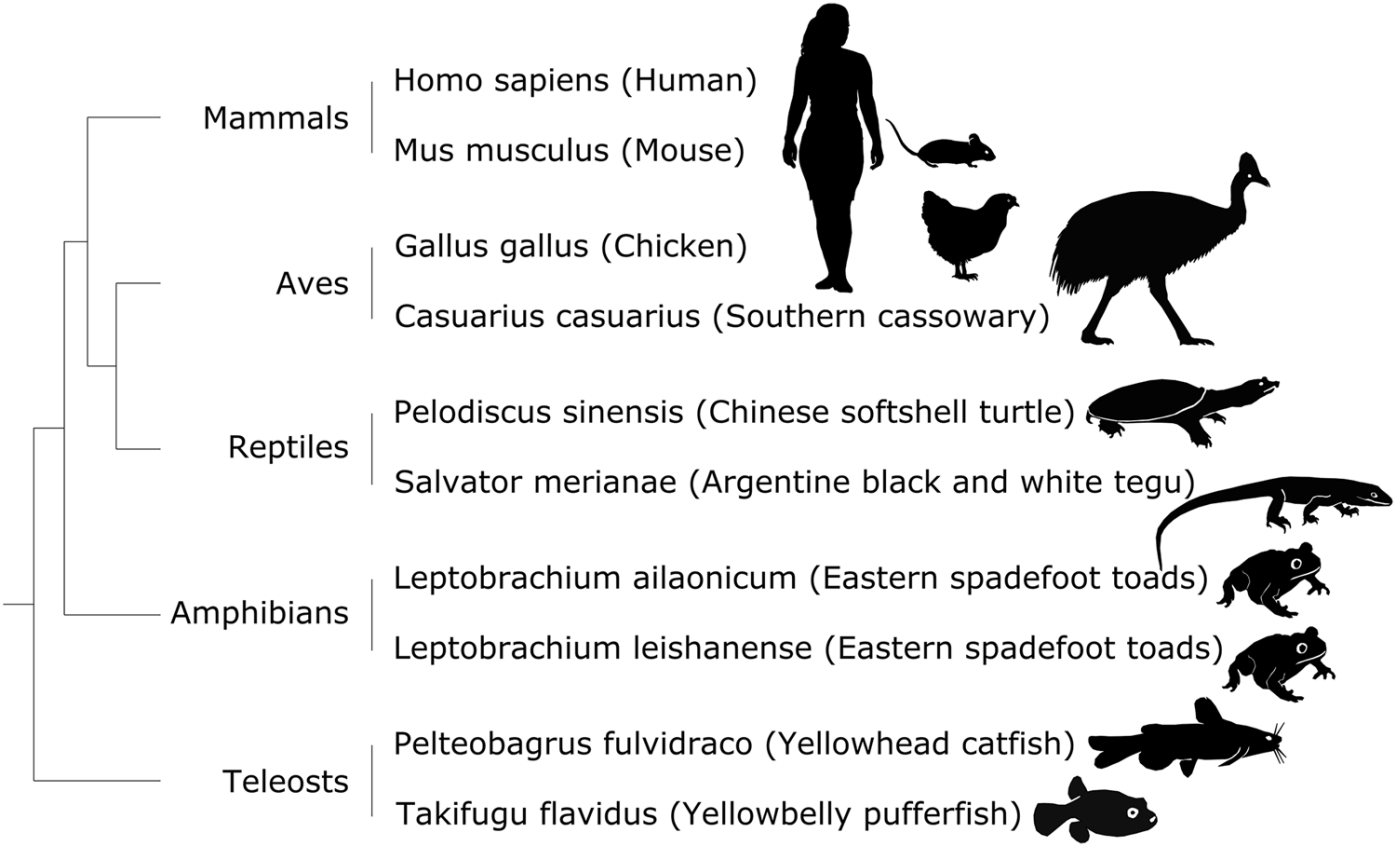
The phylogenetic tree of the species included in this study.

As a result, we collected a representative collection of Hi-C data on erythroid cells from 10 animal species belonging to the main classes of tetrapods and bony fish (Fig. 3). It is not comprehensive, especially concerning fish classes, which are a very diverse group of vertebrates, but unfortunately, there is little data available on them, therefore, they were not available for the analysis.

### Analysis of the 3D genome organization in erythroid cells of vertebrates

**Figure 4:**
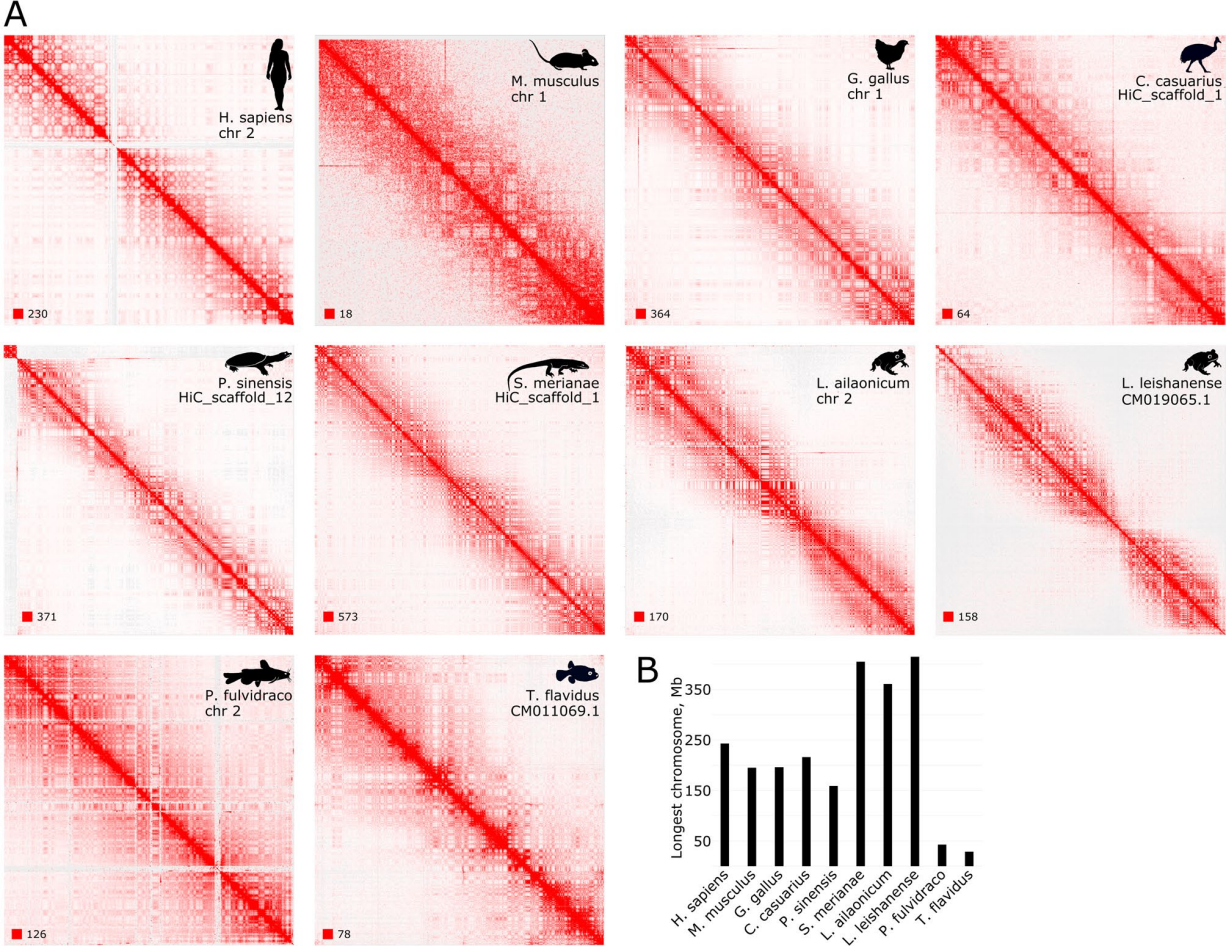
3D genome organization of erythrocytes in vertebrates. (**A**) Representative Hi-C heatmap of one of the long chromosomes for each species under analysis. (**B**) Chart of chromosome sizes depicted in (**A**). Hi-C maps were visualized with Juicebox (version 1.11.08)^7^.

We used a common pipeline to reanalyze Hi-C data collected from public sources and obtained during this study. We have generated heatmaps and plotted P(s) curves. Whole-chromosome Hi-C maps for the longer chromosomes for each of the analyzed species are shown on the Fig. 4A. The second diagonal band coexisting with the checkerboard pattern of compartments, described earlier for chicken erythrocytes’ contact map, is also represented on the heatmaps of the cassowary—a second analyzed bird species, in reptiles and amphibians. Contact maps from mouse cells demonstrate a second diagonal too, although the increase of contact probability with distance is not as pronounced as in other species. On the Hi-C maps from human erythroblasts we didn’t observe a characteristic second diagonal pattern. In both analyzed fish species nothing resembling a second diagonal was found on the heatmap.

**Figure 5:**
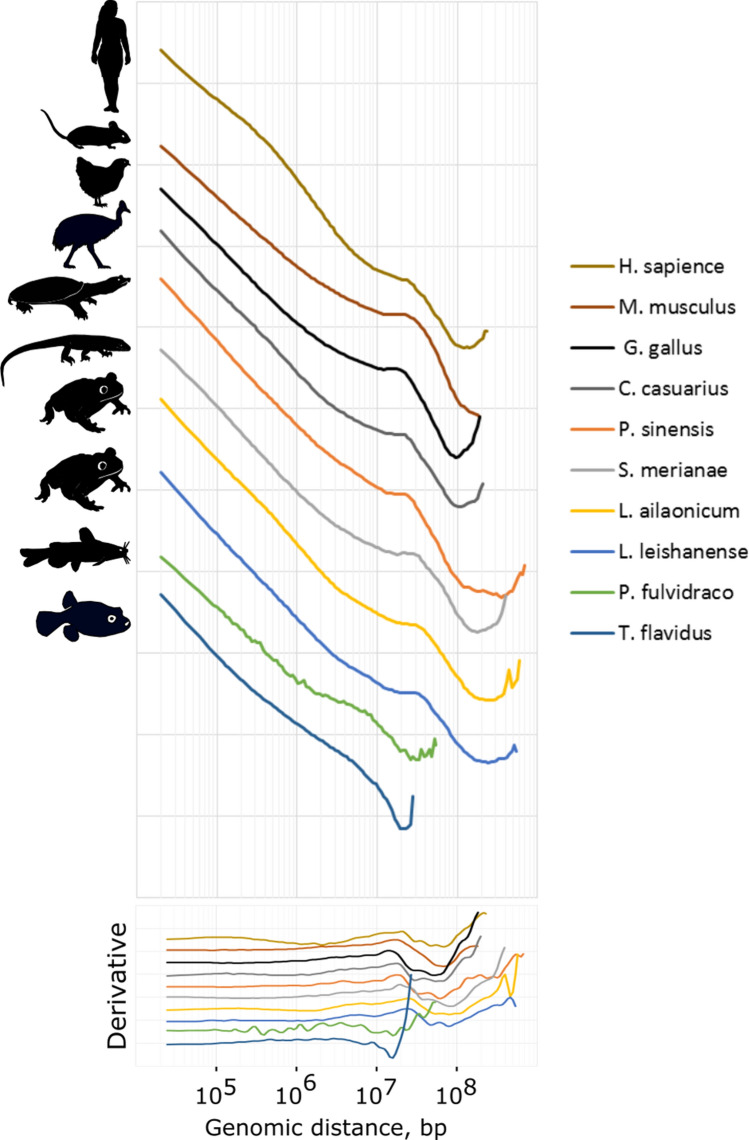
The dependence of the contact probability on the genomic distance P(s) for erythrocytes and erythroblasts (human and mouse) averaged over all chromosomes for 10 species under analysis. Bottom panel: derivatives of P(s) curves.

Analysis of the P(s) plots revealed that birds, reptiles, and amphibians demonstrate a very similar profile of contact frequencies (Fig. 5). A region between 10^5^–10^6^ bp is flat and has no upward bend, indicating the absence of TAD-level degree of compaction. The absence of typical TADs is clearly visible on the heatmaps (Supplement Fig. S1). While the genome of typical interphase cells at the sub-megabase level is characterized by the presence of structures formed by the loop extrusion mechanism (loops, stripes), in erythroid cells on the sub-megabase scale chromatin separation into A and B compartments (checkerboard pattern) dominates. Because of this peculiarity of the erythroid genome organization, the TAD callers (in particular, HiCExplorer hicFindTADs algorithm) interpret insulation at the boundaries of the compartments as TAD borders. We have also performed aggregate peak analysis (Supplement Fig. S2). The meta-loop plot revealed global disappearance of loops confirming significantly weakened domain structures.

An evident peak at the 10–30 Mb distance is consistent with the second diagonal pattern on the heatmaps. Mammalian Hi-C data from mouse ortho- and polychromatic erythroblasts produce a P(s) curve with a highly similar profile: with a local peak of long-range contact probability (~ 25 Mb) and without TAD-level compaction. However, the shape of the P(s) plot for human erythroblasts is completely different from the P(s) profile of mature erythrocytes of other tetrapods and mouse erythroblasts. It rather resembles the P(s) profile of interphase cells with typical genome organization, such as fibroblasts (Fig. 1B). First, they have a clear curvature on the P(s) plot corresponding to the characteristic TAD length. Indeed, loops and stripes are clearly visible on human erythroblast Hi-C maps—structural features that we do not observe in mature erythrocytes of other animals. Next, these cells do not display a specific pattern of interactions seen as a second diagonal on Hi-C maps (Fig. 5). The point is, that in Huang et al. a population of basophilic erythroblasts was used for the Hi-C experiment. At this differentiation stage, chromatin compaction and decrease in the nucleus size are not so pronounced, and specific genome organization is not fully formed. Therefore, on the heatmap of human erythroblasts we most likely see a 3D genome organization typical for dividing cells.

To independently confirm the distinctive features of 3D genome organization in erythroid cells in mammals, we added the analysis of Hi-C data from Oudelaar et al.^3^. The authors used murine Ter119 + cells from the spleen. As can be seen from Supplementary Figure S3, the contact maps from these cells are also characterized by the second diagonal band and the absence of TADs. Therefore, although we do not have suitable data on human erythroid cells, our observations are supported by two sets of independent data from murine erythroblasts, which makes the assumption of a conservative 3D genome organization in mammalian erythroblasts more reasonable.

For both analyzed fish species, we detect no signs of the second diagonal pattern either on the Hi-C maps or on the P(s) curves (Figs. 4A, 5). However, in agreement with the data from mature erythrocytes of other analyzed species, fish RBCs lack TADs and other loop extrusion-associated structures.

Thus, we conclude that the absence of TADs in the genome of mature erythrocytes in non-mammalian vertebrates and late erythroblasts in mammals is an evolutionary conservative feature. In addition, the genome of terminally differentiated erythroid cells in amphibians, reptiles, birds and mammals is characterized by an increased contact frequency at a distance of 10–30 Mb.

## Discussion

Current study demonstrates that the features of the 3D genome organization of mature erythrocytes, described earlier in chickens, are conservative for vertebrates. Apparently, this represents nuclear condensation and transcriptional silencing, which is a common process for erythrocyte maturation in different species^9^. However, the corresponding molecular mechanisms governing reorganization in the erythroid genome structure have yet to be clarified. It is known that TADs in interphase chromosomes are formed by the mechanism of active loop extrusion^10^. The chromatin loop is stretched through a ring shaped cohesin complex until this process is stopped by two convergently oriented CTCF binding sites, forming the loop anchor. It was previously shown that both the cohesin subunit Rad21 and CTCF protein are present in the nuclei of mature chicken erythrocytes, although they tend to form unusual clumps^6,11^. Therefore, it remains unclear whether these key loop extrusion factors remain bound to chromatin or are displaced from chromatin fiber and thus are non-functional.

An alternative explanation for the disappearance of TADs could be a general decrease of the metabolic activity in erythrocytes^12^. Goto et al. has revealed that intracellular ATP level decreases in erythroblasts undergoing terminal differentiation. Indeed, during erythroid differentiation, multiple events occur that require significant consumption of ATP: breakdown of the nuclear pore complex and redistribution of its components, global histone remodeling and deacetylation, export of many nuclear proteins, polarization of the condensed nucleus, mediated by rearrangement of microtubules associated with dynein, sorting of membrane proteins, programmed death of organelles and nuclear protein degradation^13–19^. It is known that ATP hydrolysis is required for the loop extrusion by cohesin^20^. Therefore, it is possible that in the absence of energy from ATP hydrolysis cohesin loading and subsequent loop extrusion process stop in mature erythroid cells. However, more data is needed to test this theory.

ATP dynamics can also affect chromatin structure in a different way. The effect of decreased level of cellular ATP was studied both on mitotic chromosomes and in the interphase nucleus revealing an increase in chromatin compaction following the decrease in ATP level^21,22^. Maeshima et al. has demonstrated that Mg2 + is released from ATP-Mg2 + complexes by ATP hydrolysis and facilitates mitotic chromosome condensation^23^. Besides there is an influx of Ca2 + into the late erythroblasts^24^. Free divalent cations were shown to condense chromosomes in vitro, suggesting that increased Ca2 + might also affect erythroid chromatin condensation.

Another interesting feature of the 3D chromatin organization is an increased frequency of contacts at a genomic distance of 10–30 Mb. A similar characteristic was previously described for mitotic chromosomes in chicken and human cells. Gibcus et al. suggest that in mitotic chromosomes an increase in contact frequencies, occurring at ~ 12 Mb distance, is due to a specific mechanism of chromosome compaction, independent of TADs and compartments. Prometaphase chromosomes are folded into helical arrangement of condensin-dependent loop arrays, to achieve axial shortening and density. The pattern of second diagonal on Hi-C maps corresponds to the increased frequency of contacts between the loci located one turn up or down the helix. Upon G1 entry, chromosome maps from synchronous DT40 and HeLa cell cultures no longer display the second diagonal pattern of contacts. Apparently, this complicated loop array structure gradually resolves in cells by G1 phase^5^. Mature nucleated vertebrate erythrocytes are non-proliferating cells on the G1 phase^25^. We assume that the second diagonal pattern of the Hi-C contacts, which is completely uncommon for an interphase nucleus, could be inherited from metaphase chromosomes. It is possible that the helical arrangement of loops in the nuclei of erythrocytes persists up to the G1 stage. This may be supported by the fact that condensin is especially abundant in erythroblasts^26^. It is not yet clear what is the functional role of such an extraordinary genome organization in erythrocytes. We can speculate that, as for mitotic chromosomes, this could be a higher degree of genome compaction.

Notably, we found no evidence of a second diagonal in the analyzed bony fish species. It should be noted that considering the short size of fish chromosomes, detection of an increased frequency of contacts at a 10–30 Mb distance seems impossible. This is clearly seen from Fig. 4B, which shows the ratio of the sizes for the longest chromosomes in all analyzed species. Thus, it is quite possible that the chromosomes in fish erythrocytes do have an ordered helical loop structure characteristic of other vertebrates, but this does not lead to an increase in the frequency of distant (more than 10 Mb) contacts due to the length of chromosomes.

In general, our study reveals conservation of 3D genome organization principles in vertebrate erythroid cells, which are characterized by a highly compact and transcriptionally inactive nucleus.

## Materials and methods

### Mouse erythroblasts preparation

The mice were maintained on a 12‐hour light/dark cycle with ad libitum food and water in a conventional animal facility^27^. All experiments were conducted at the Department of Experimental Animal Genetic Resources at the Institute of Cytology and Genetics, SB RAS (RFMEFI61914X0005 and FMEFI61914X0010). All the procedures and technical manipulations with animals were in compliance with the European Communities Council Directive of 24 November 1986 (86/609/EEC) and approved by the Bioethics Review Committee of the Institute of Cytology and Genetics (Permission N45 from November 16, 2018).

Bone marrow was isolated from the tibias and femurs of C57Bl/6 mice according to^28^. Bone marrow cells were dissociated by gentle pipetting in PBS + 0.2% BSA and passed through a 70 µm cell strainer. Cells were washed once in ice-cold staining buffer (PBS, 0.2% BSA, 5% FBS) for further FACS staining. Bone marrow cell suspension was pre-blocked with CD16/CD32 (BioLegend) for 20 min and subsequently stained with FITC-conjugated anti-CD44 (BioLegend) and PE-conjugated anti-TER119 (BioLegend) for 1 h at 4 °C in the dark. At the end of incubation, cells were washed twice in ice-cold staining buffer (300 g for 5 min) and resuspended in 1 ml of staining buffer for cell sorting. Unstained cells were used as a negative control. To isolate erythroblasts at poly- and orthochromatic stages we used the gating strategy from^29^ with some modifications.

### In situ Hi-C

Hi-C experiment was performed using previously published protocol^30^. Approximately 6*10^5^ cells were fixed with 1% PFA (Sigma-Aldrich) for a replicate, a total of 3 replicates for each differentiation stage (polychromatic and orthochromatic). After cell lysis and nuclear permeabilization, chromatin was digested with 100 U of DpnII (NEB). Restriction fragments overhangs were filled in with biotin-dCTP at 37 °C. After proximity ligation and reversal of crosslinks, DNA was purified and sheared on a Covaris sonicator (LE220, Covaris, Woburn, MA) to a length of 200–400 bp. Biotinylated ligation products were then pulled down with Dynabeads MyOne Streptavidin C1 (Invitrogen) and prepared for sequencing with NEBNext Ultra II DNA Library Prep Kit for Illumina. Libraries were sequenced on an Illumina HiSeq 2500 system (2 × 150 bp, paired-end). After the reproducibility of replicates was confirmed by the pairwise stratum-adjusted correlation coefficients count (Supplementary Fig. S4), data from all replicas was merged.

### Computational methods

Sequencing data and genome assemblies produced by other studies^6,8,31–36^ were downloaded from public resources listed in the Supplementary Table S1. Fastq files were extracted from the SRA archives using fastq-dump. Next, a list of Hi-C contacts was obtained using Juicer tool software (version 1.5.6)^37^. Juicer output was processed with cooler software (version 0.8.7)^38^. First, data were converted to cool format and binned at 10 kb resolution using cload pairs module, then matrixes were built and interactively corrected using balance module. Hi-C analysis statistics for all data are provided in Supplementary Table S2.

We selected chromosomes (or scaffolds when the chromosome-level assembly was not available) longer than 9 Mb and computed the contact frequency (P) as a function of genomic distance (s), as well as corresponding derivative values using cooltools expected function (https://github.com/mimakaev/cooltools/blob/master/cooltools/). Resolution in 10 bins per order magnitude was used for plots.

To call compartments, PC1 values of Hi-C matrices were computed at the resolution of 50 Kb using Juicer tools eigenvector module^37^. TADs were called at the resolution of 10 kb using HiCExplorer hicFindTADs function with delta value equals to 0.05^39–41^.

The plots with aggregate contact enrichment at the corner of TAD were generated using coolpup.py tool at the 10 kb resolution^42^. First, we provided .bedpe file with coordinates of TADs’ corners + -10 kb to coolpup command. After that we used plotpup command with vmin = 0.3 and vmax = 3.

The pairwise stratum-adjusted correlation coefficients were computed for matrices at the 100 Kb resolution using hicrep’s algorithm^43^. Smoothing parameter h equals to 3 and maximum distance equals to 5 Mb were used.

## Supplementary Information

Supplementary Information 1.

Supplementary Information 2.

Supplementary Information 3.

## Data Availability

The raw Hi-C sequencing data of mouse erythroblasts were deposited in the SRA under Bioproject No. PRJNA666472.

## References

[1] Bianconi E (2013). An estimation of the number of cells in the human body. Ann. Hum. Biol..

[2] Mei Y, Liu Y, Ji P (2020). Understanding terminal erythropoiesis: An update on chromatin condensation, enucleation, and reticulocyte maturation. Blood Rev..

[3] Oudelaar AM (2018). Single-allele chromatin interactions identify regulatory hubs in dynamic compartmentalized domains. Nat. Genet..

[4] Chiariello AM (2020). A dynamic folded hairpin conformation is associated with α-globin activation in erythroid cells. Cell Rep..

[5] Gibcus JH (2018). A pathway for mitotic chromosome formation. Science (80-).

[6] Fishman V (2019). 3D organization of chicken genome demonstrates evolutionary conservation of topologically associated domains and highlights unique architecture of erythrocytes’ chromatin. Nucleic Acids Res..

[7] Durand NC (2016). Juicebox provides a visualization system for Hi-C contact maps with unlimited zoom. Cell Syst..

[8] Huang P (2017). Comparative analysis of three-dimensional chromosomal architecture identifies a novel fetal hemoglobin regulatory element. Genes Dev..

[9] Ji P, Murata-Hori M, Lodish HF (2011). Formation of mammalian erythrocytes: Chromatin condensation and enucleation. Trends Cell Biol..

[10] Mirny LA, Imakaev M, Abdennur N (2019). Two major mechanisms of chromosome organization. Curr. Opin. Cell Biol..

[11] Kantidze OL, Iarovaia OV, Philonenko ES, Yakutenko II, Razin SV (2007). Unusual compartmentalization of CTCF and other transcription factors in the course of terminal erythroid differentiation. Biochim. Biophys. Acta Mol. Cell Res..

[12] Goto T (2019). ATP produced by anaerobic glycolysis is essential for enucleation of human erythroblasts. Exp. Hematol..

[13] Wang J (2012). Mammalian erythroblast enucleation requires PI3K-dependent cell polarization. J. Cell Sci..

[14] Kobayashi I (2016). Erythroblast enucleation is a dynein-dependent process. Exp. Hematol..

[15] Zhao B (2016). Nuclear condensation during mouse erythropoiesis requires caspase-3-mediated nuclear opening. Dev. Cell.

[16] Hattangadi SM (2014). Histones to the cytosol: Exportin 7 is essential for normal terminal erythroid nuclear maturation. Blood.

[17] Figueroa AA (2018). MiR-181a regulates erythroid enucleation via the regulation of Xpo7 expression. Haematologica.

[18] Wilkins BJ (2014). A cascade of histone modifications induces chromatin condensation in mitosis. Science (80-).

[19] Zhen R (2020). Wdr26 regulates nuclear condensation in developing erythroblasts. Blood.

[20] Kim Y, Shi Z, Zhang H, Finkelstein IJ, Yu H (2019). Human cohesin compacts DNA by loop extrusion. Science (80-)..

[21] Visvanathan A (2013). Modulation of higher order chromatin conformation in mammalian cell nuclei can be mediated by polyamines and divalent cations. PLoS ONE.

[22] Nozaki T (2017). Dynamic organization of chromatin domains revealed by super-resolution live-cell imaging. Mol. Cell.

[23] Maeshima K (2018). A transient rise in free Mg2+ ions released from ATP-Mg hydrolysis contributes to mitotic chromosome condensation. Curr. Biol..

[24] Wölwer CB, Pase LB, Russell SM, Humbert PO (2016). Calcium signaling is required for erythroid enucleation. PLoS ONE.

[25] Dolznig, H., Bartunek, P., Nasmyth, K., Mullner, E. W. & Beug, H. Terminal differentiation of normal chicken erythroid progenitors: Shortening of G1 correlates with loss of D-cyclin/cdk4 expression and altered cell size control. *Cell Growth Differ.* (1995). 8562472

[26] Xu Y, Leung CG, Lee DC, Kennedy BK, Crispino JD (2006). MTB, the murine homolog of condensin II subunit CAP-G2, represses transcription and promotes erythroid cell differentiation. Leukemia.

[27] Korablev A, Lukyanchikova V, Serova I, Battulin N (2020). On-target CRISPR/Cas9 activity can cause undesigned large deletion in mouse zygotes. Int. J. Mol. Sci..

[28] Madaan A, Verma R, Singh AT, Jain SK, Jaggi M (2014). A stepwise procedure for isolation of murine bone marrow and generation of dendritic cells. J. Biol. Methods.

[29] Liu J (2013). Quantitative analysis of murine terminal erythroid differentiation in vivo: Novel method to study normal and disordered erythropoiesis. Blood.

[30] Belaghzal H, Dekker J, Gibcus JH (2017). Hi-C 2.0: An optimized Hi-C procedure for high-resolution genome-wide mapping of chromosome conformation. Methods.

[31] Li Y (2019). Chromosome-level assembly of the mustache toad genome using third-generation DNA sequencing and Hi-C analysis. Gigascience.

[32] Gong G (2018). Chromosomal-level assembly of yellow catfish genome using third-generation DNA sequencing and Hi-C analysis. Gigascience.

[33] Roscito JG (2018). The genome of the tegu lizard Salvator merianae: Combining Illumina, PacBio, and optical mapping data to generate a highly contiguous assembly. Gigascience.

[34] Dudchenko O (2017). De novo assembly of the Aedes aegypti genome using Hi-C yields chromosome-length scaffolds. Science (80-).

[35] Dudchenko O (2018). The juicebox assembly tools module facilitates <em>de novo</em> assembly of mammalian genomes with chromosome-length scaffolds for under $1000. bioRxiv.

[36] Kadota M (2020). Multifaceted Hi-C benchmarking: What makes a difference in chromosome-scale genome scaffolding?. Gigascience.

[37] Durand NC (2016). Juicer provides a one-click system for analyzing loop-resolution Hi-C experiments. Cell Syst..

[38] Kerpedjiev P (2018). HiGlass: Web-based visual exploration and analysis of genome interaction maps. Genome Biol..

[39] Wolff J (2020). Galaxy HiCExplorer 3: A web server for reproducible Hi-C, capture Hi-C and single-cell Hi-C data analysis, quality control and visualization. Nucleic Acids Res..

[40] Wolff J (2018). Galaxy HiCExplorer: a web server for reproducible Hi-C data analysis, quality control and visualization. Nucleic Acids Res..

[41] Ramírez F (2018). High-resolution TADs reveal DNA sequences underlying genome organization in flies. Nat. Commun..

[42] Flyamer IM, Illingworth RS, Bickmore WA (2020). Coolpup.py: Versatile pile-up analysis of Hi-C data. Bioinformatics.

[43] Yang T (2017). HiCRep: Assessing the reproducibility of Hi-C data using a stratum-adjusted correlation coefficient. Genome Res..

