# Supplementary material for: Erythrocytes 3D genome organization in vertebrates
Source: Sci Rep. 2021 Feb 24;11:4414. doi: 10.1038/s41598-021-83903-9 (PMC7904930; doi:10.1038/s41598-021-83903-9)
Supplement: Supplementary file 1 — Supplementary Information 1. [file 41598_2021_83903_MOESM1_ESM.pdf]

Supplementary Materials for

Title: Erythrocytes 3D genome organization in vertebrates

Anastasia Ryzhkova[1], Alena Taskina[2], Anna Khabarova[1], Veniamin Fishman[1,2], Nariman Battulin[1,2]

1. Institute of Cytology and Genetics SB RAS, Novosibirsk, Russia
2. Novosibirsk State University, Novosibirsk, Russia

Corresponding author: Nariman Battulin
battulin@gmail.com

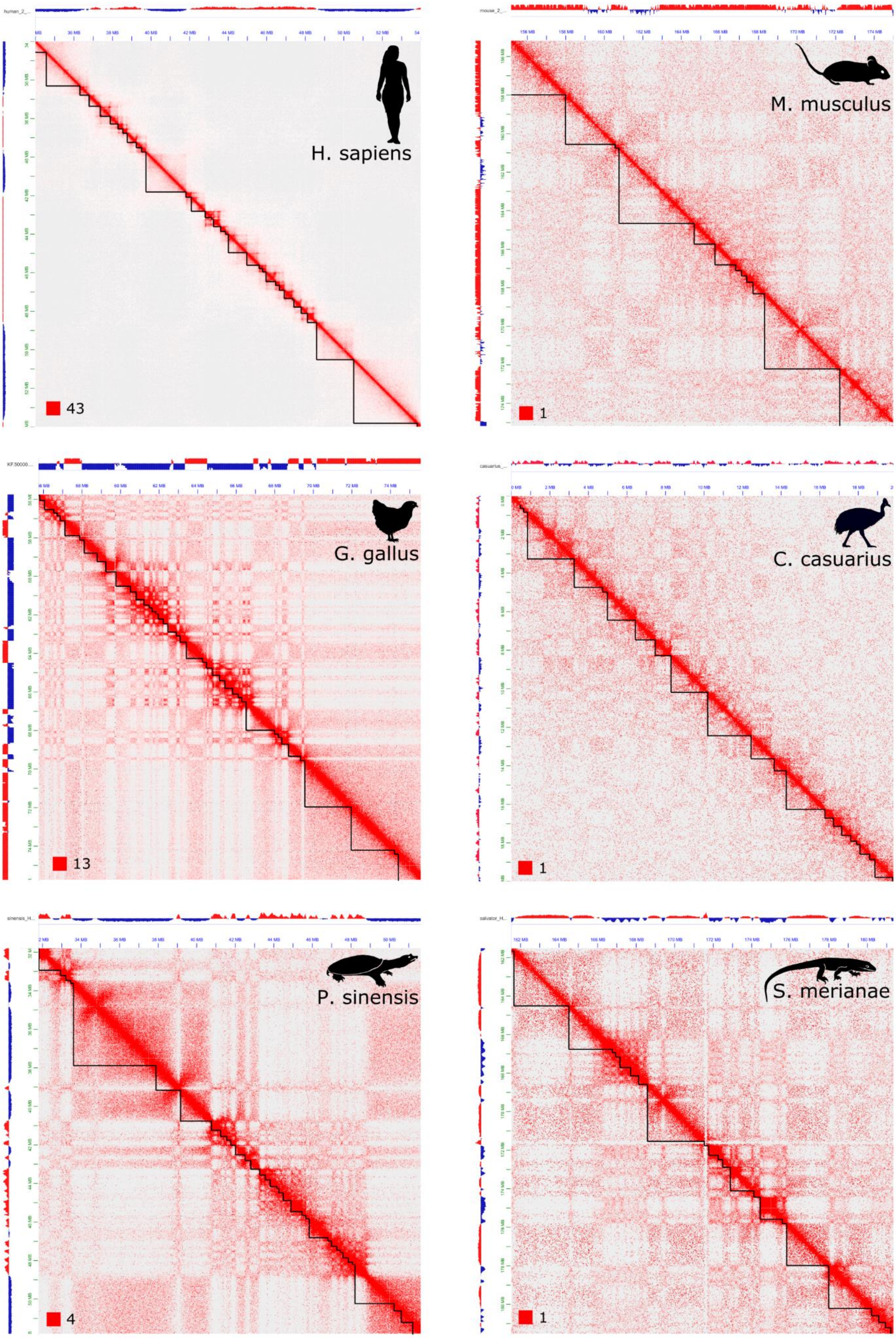
H. sapiens
43
M. musculus
1
G. gallus
13
C. casuarius
1
P. sinensis
4
S. merianae
1

Supplementary Figure S1 (continued)

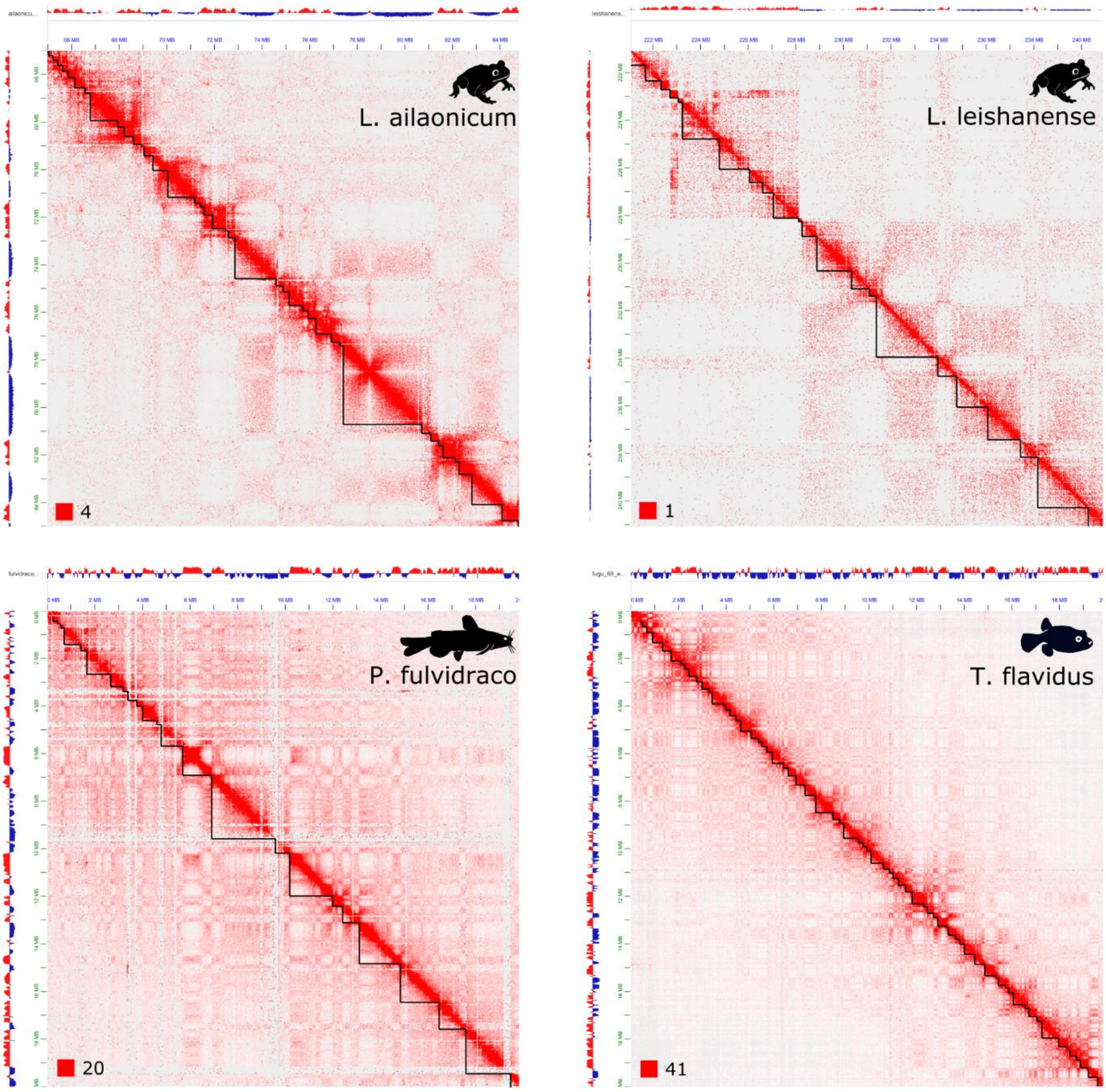

Supplementary Figure S1. Examples of Hi-C heat maps of erythroid cells. Randomly selected 38 Mb region at 25 kb resolution are depicted. Black triangles under the diagonal are TADs called by HiCExplorer hicFindTADs algorithm. Red and blue tracks are PC1 values of Hi-C matrices. Hi-C maps were visualized with Juicebox (version 1.11.08).

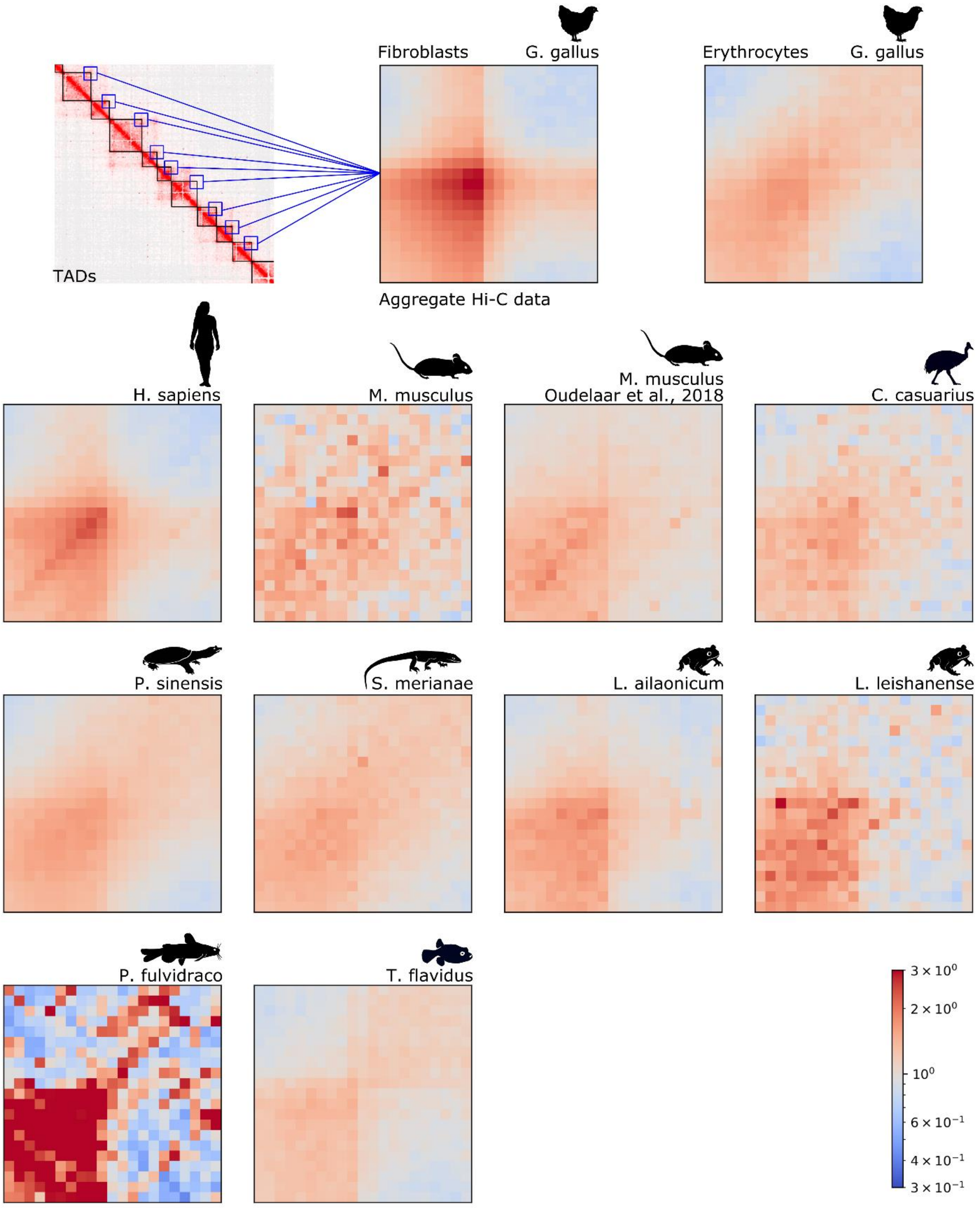

Supplementary Figure S2. TADs identified by TADcallers in erythroid cells differ from typical TADs. Aggregate Hi-C data binned at 10 kb resolution at TAD peak identified by HiCExplorer hicFindTADs algorithm. Chicken fibroblasts are presented as an example of typical interphase cells. Strong enrichment at the center indicates that TADs in fibroblasts have increased contacts between the borders. In all other analyzed datasets except for human erythroblasts no such enrichment present. Aggregate Hi-C analysis for P. fulvidraco appears to be different due to imperfect genomic assembly for this species.

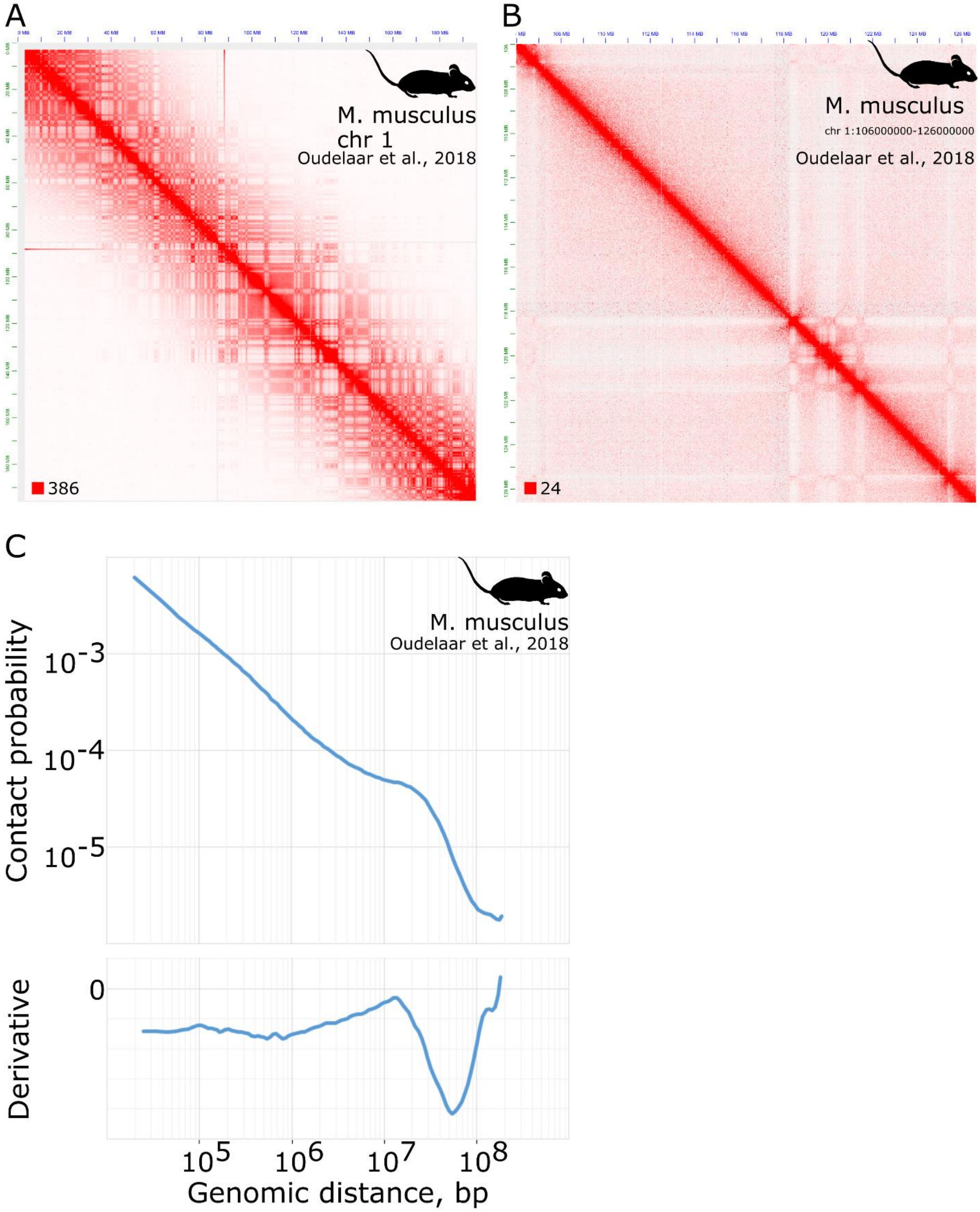

Supplementary Figure S3. 3D genome organization of mouse splenic Ter-119+ cells (data from Oudelaar et al., 2018). **A**. Whole chromosome heat map of chromosome 1. **B**. Randomly selected region at 25 kb resolution. **C**. The dependence of the contact probability on the genomic distance P(s) averaged over all chromosomes. Bottom panel: derivative of P(s) curve. Hi-C maps were visualized with Juicebox (version 1.11.08).

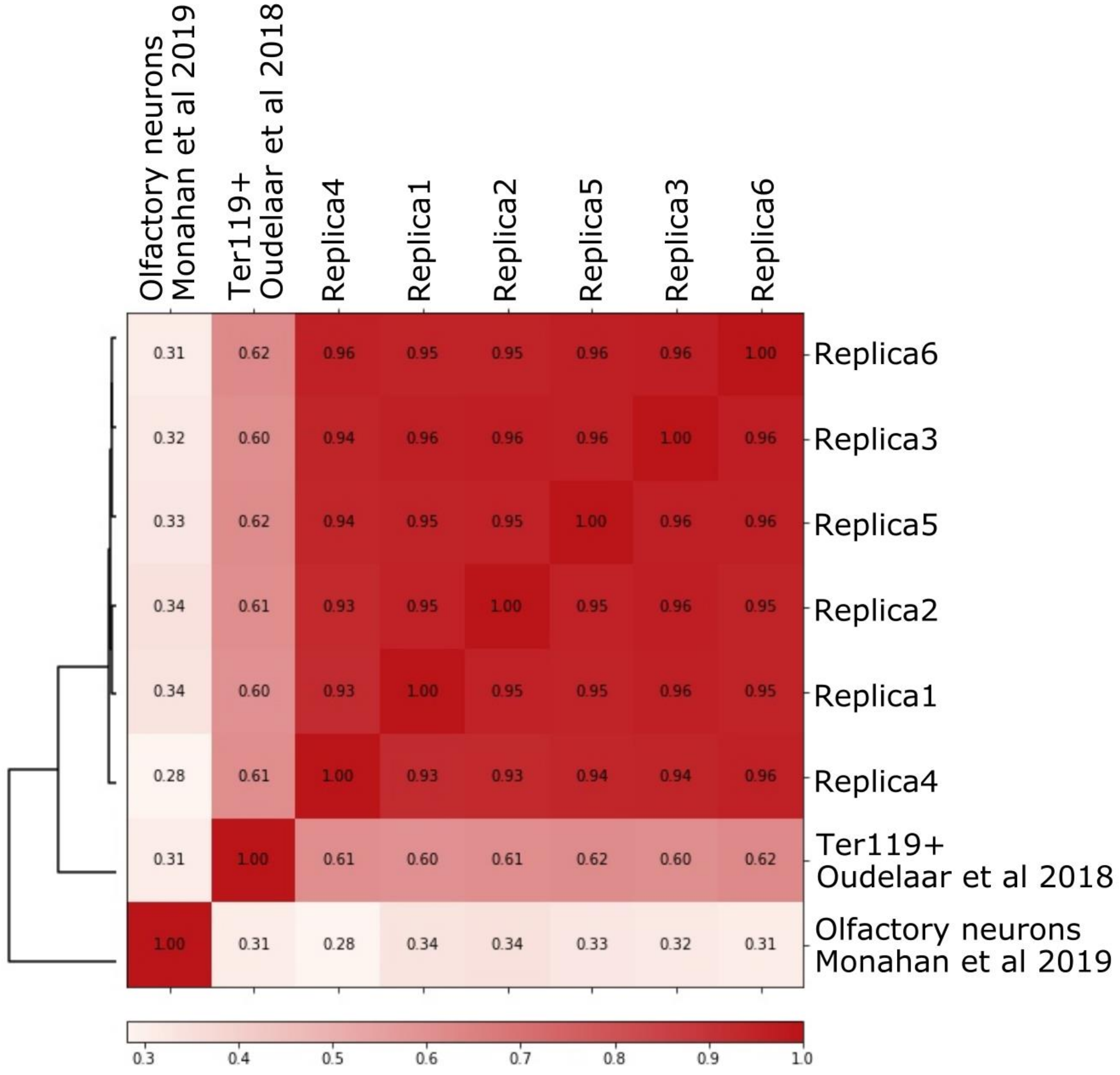

Supplementary Figure S4. The pairwise stratum-adjusted correlation coefficients for mouse erythroblasts Hi-C replicas and two other cell types for comparison.
