# Supplementary material for: Erythrocytes 3D genome organization in vertebrates
Source: Sci Rep. 2021 Feb 24;11:4414. doi: 10.1038/s41598-021-83903-9 (PMC7904930; doi:10.1038/s41598-021-83903-9)
Supplement: Supplementary file 2 — Supplementary Information 2. [file 41598_2021_83903_MOESM2_ESM.docx]

**Supplementary Materials for**

**Title: Erythrocytes 3D genome organization in vertebrates**

Anastasia Ryzhkova^1^, Alena Taskina^2^, Anna Khabarova^1^, Veniamin Fishman^1,2^, Nariman Battulin^1,2^

1. Institute of Cytology and Genetics SB RAS, Novosibirsk, Russia

2. Novosibirsk State University, Novosibirsk, Russia

Corresponding author: Nariman Battulin

battulin@gmail.com

**Supplementary Table 1. Sequencing data and genome assemblies used in this study**

| Species | Tissue | Hi-C reads | Genome | References* |
| --- | --- | --- | --- | --- |
| *Leptobrachium leishanense* | Blood | SRX5574638-SRX5574645 | GCA_009667805.1 (GeneBank) |  |
| *Leptobrachium ailaonicum* | Blood | SRX5514057 | ftp://parrot.genomics.cn/gigadb/pub/10.5524/100001_101000/100624/Leptobrachium_ailaonicumlaonica_genome.chromosome.fa.gz | Li et al., 2019 ^1^ |
| *Takifugu flavidus* | Blood | SRX4720189 | GCA_003711565.2  (GeneBank) |  |
| *Pelteobagrus fulvidraco* | Blood | SRX4669750 | ftp://parrot.genomics.cn/gigadb/pub/10.5524/100001_101000/100506/P.fulvidraco.female.genome.fasta | Gong et al., 2018 ^2^ |
| *Salvator merianae* | Blood | SRX5415917 | <https://www.dropbox.com/s/x6vut6u55osw7pt/HLtupMer6_HiC.fasta.gz?dl=0> | DNA ZOO (Roscito et al., 2018; Dudchenko et al., 2017, 2018)^3–5^ |
| *Pelodiscus sinensis* | Blood | DRX167734  DRX167732 | ftp://parrot.genomics.cn/gigadb/pub/10.5524/100001_101000/100675/scaffolding_results/ID8_scaffolding.tar.gz | Kadota et al., 2020 ^6^ |
| *Casuarius casuarius* | Blood | SRX7041748 | casCas1  GCA_003342895.1 | DNA ZOO (Roscito et al., 2018; Dudchenko et al., 2017, 2018) ^4,5,7^ |
| *Gallus gallus* | Mature erythrocytes | SRX2629741  SRX2629742 | galGal5  GCA_000002315.3 | Fishman et al., 2019 ^8^ |
| *Mus musculus* | Ortochromatic and polychromatic erythroblasts from bone marrow | PRJNA666472 | GRCm38  GCA_000001635.2 | This study |
| *Mus musculus* | Splenic ter119+ cells | SRX3461898  SRX3461899 | GRCm38  GCA_000001635.2 | Oudelaar et al., 2018^9^ |
| *Homo sapiens* | Adult erythroblasts | SRX3058043  SRX3058042 | GRCh38  GCA_000001405.15 | Huang et al., 2017 ^10^ |

***References**

1. Li, Y. *et al.* Chromosome-level assembly of the mustache toad genome using third-generation DNA sequencing and Hi-C analysis. *Gigascience* (2019). doi:10.1093/gigascience/giz114

2. Gong, G. *et al.* Chromosomal-level assembly of yellow catfish genome using third-generation DNA sequencing and Hi-C analysis. *Gigascience* (2018). doi:10.1093/gigascience/giy120

3. Roscito, J. G. *et al.* The genome of the tegu lizard Salvator merianae: combining Illumina, PacBio, and optical mapping data to generate a highly contiguous assembly. *Gigascience* (2018). doi:10.1093/gigascience/giy141

4. Dudchenko, O. *et al.* De novo assembly of the Aedes aegypti genome using Hi-C yields chromosome-length scaffolds. *Science (80-. ).* (2017). doi:10.1126/science.aal3327

5. Dudchenko, O. *et al.* The Juicebox Assembly Tools module facilitates &lt;em&gt;de novo&lt;/em&gt; assembly of mammalian genomes with chromosome-length scaffolds for under $1000. *bioRxiv* 254797 (2018). doi:10.1101/254797

6. Kadota, M. *et al.* Multifaceted Hi-C benchmarking: What makes a difference in chromosome-scale genome scaffolding? *Gigascience* (2020). doi:10.1093/gigascience/giz158

7. Sackton, T. B. *et al.* Convergent regulatory evolution and loss of flight in paleognathous birds. *Science (80-. ).* (2019). doi:10.1126/science.aat7244

8. Fishman, V. *et al.* 3D organization of chicken genome demonstrates evolutionary conservation of topologically associated domains and highlights unique architecture of erythrocytes’ chromatin. *Nucleic Acids Res.* **47**, 648–665 (2019).

9. Oudelaar, A. M. *et al.* Single-allele chromatin interactions identify regulatory hubs in dynamic compartmentalized domains. *Nat. Genet.* (2018). doi:10.1038/s41588-018-0253-2

10. Huang, P. *et al.* Comparative analysis of three-dimensional chromosomal architecture identifies a novel fetal hemoglobin regulatory element. *Genes Dev.* (2017). doi:10.1101/gad.303461.117
